# Advances in immunotherapy for treatment of lung cancer

**DOI:** 10.7497/j.issn.2095-3941.2015.0032

**Published:** 2015-09

**Authors:** Jean G. Bustamante Alvarez, María González-Cao, Niki Karachaliou, Mariacarmela Santarpia, Santiago Viteri, Cristina Teixidó, Rafael Rosell

**Affiliations:** ^1^Albert Einstein Medical Center, Philadelphia 19141, USA; ^2^Translational Cancer Research Unit, Instituto Oncológico Dr Rosell, Quirón Dexeus University Hospital, Barcelona 08028, Spain; ^3^Medical Oncology Unit, Human Pathology Department, University of Messina, Messina 98100, Italy; ^4^Pangaea Biotech S.L, Barcelona 08028, Spain; ^5^Cancer Biology & Precision Medicine Program, Catalan Institute of Oncology, Germans Trias i Pujol Health Sciences Institute and Hospital, Campus Can Ruti, Badalona, Barcelona 08916, Spain; ^6^Fundación Molecular Oncology Research, Barcelona 08028, Spain

**Keywords:** Cytotoxic T-lymphocyte-associated protein 4 (CTLA-4), immune checkpoint inhibitors, lung cancer, programmed cell death protein ligand-1 (PD-L1), programmed cell death protein 1 (PD-1)

## Abstract

Different approaches for treating lung cancer have been developed over time, including chemotherapy, radiotherapy and targeted therapies against activating mutations. Lately, better understanding of the role of the immunological system in tumor control has opened multiple doors to implement different strategies to enhance immune response against cancer cells. It is known that tumor cells elude immune response by several mechanisms. The development of monoclonal antibodies against the checkpoint inhibitor programmed cell death protein 1 (PD-1) and its ligand (PD-L1), on T cells, has led to high activity in cancer patients with long lasting responses. Nivolumab, an anti PD-1 inhibitor, has been recently approved for the treatment of squamous cell lung cancer patients, given the survival advantage demonstrated in a phase III trial. Pembrolizumab, another anti PD-1 antibody, has received FDA breakthrough therapy designation for treatment of non-small cell lung cancer (NSCLC), supported by data from a phase I trial. Clinical trials with anti PD-1/PD-L1 antibodies in NSCLC have demonstrated very good tolerability and activity, with response rates around 20% and a median duration of response of 18 months.

## Introduction

Efforts in the development of therapy for metastatic lung cancer have led to an evolving arsenal of different approaches including chemotherapy and molecular targeted therapies. However, metastatic lung cancer remains the leading cause of cancer death worldwide and 5-year survival for advanced disease is less than 5%.

Multiple studies have shown how post-transplant patients undergoing immunosuppressive treatment have increased incidence of different types of cancer, including lymphoproliferative disorders, head and neck and lung cancer, illustrating how immunosuppression is involved in cancer development^1^^,^^2^. By means of different immunosuppressive mechanisms, including “immune check points”, lung cancer manages to evade immunosurveillance. These immune checkpoints are receptors expressed on T cells that regulate the immune response. The first immune checkpoints described were cytotoxic T-lymphocyte-associated protein 4 (CTLA-4) and the programmed cell death protein 1 (PD-1) and its ligand (PD-L1)^3^.

CTLA-4 is expressed on the surface of T-cells and regulates the amplitude of T-cell activation, down-modulates T helper cell activity and enhances regulatory T (Tregs) cell immunosuppressive activity^4^. PD-1 receptor is an inhibitory molecule present in T activated cells, B cells, monocytes, and natural killer (NK) cells. This receptor binds to its ligand PD-L1 (or B7-H1 or CD274) and PD-L2 (or B7 DC or CD273) which are expressed in tumor cells and antigen presenting cells. Up to 57% of non-small cell lung cancers (NSCLCs) express PD-L1 constitutively or as an acquired adaptive mechanism of immune resistance. Expression of tumor PD-L1 protein in NSCLC was associated with increased local lymphocytic infiltration and longer overall survival (OS)^5^. PD-1 acts within the tumor microenvironment peripherally^6^, inhibiting T-cell signaling, cytotoxic activity, proliferation, survival and effector function of T cells and also promoting differentiation of CD4^+^ T cells into T regulatory cells and inducing T cell apoptosis^6^.

In view of these immunosuppressive mechanisms, clinical research in the last decade has encompassed targeting these immune check points using monoclonal antibodies like ipilimumab, an anti CTLA-4, and pembrolizumab and nivolumab against PD-1^7^^,^^8^. These promising monoclonal antibodies were initially approved for metastatic melanoma and now several PD-1/PD-L1 inhibitors have recently been approved by the FDA also for lung cancer.

Antigen presenting cells present peptides to tumor activated specific T lymphocytes through the major histocompatibility complex class I^9^^,^^10^. Cancer cells also augment secretion of gamma interferon (INF-γ) and tumor necrosis factor α (TNF-α) by CD4^+^ T helper lymphocytes (TH)^10^. High intratumoral T cell density is required to eliminate cancer cells. Prognosis can be affected by the quantity, localization, and phenotype of infiltrating T cells^11^; high infiltration by cytotoxic T cells confers good prognosis^12^. It is also important to consider that some other inhibitory cells infiltrate tumors, like regulatory T CD4^+^ cells FOXP3^+^ (Tregs), cancer associated fibroblasts, myeloid derived suppressor cells and tumor associated macrophages^13^. These cells generate an immunosuppressive microenvironment by several mechanisms, such as TGF-β and interleukin-10 secretion, secretion of platelet derived growth factor (PDGF) and vascular endothelial growth factor (VEGF). Tumor cells, on the other hand, produce down-regulation of the major histocompatibility complex class I (MHC-I) and antigen expression and increase PD-L1 expression in tissue. As a result, solid tumors attain an immunological response insufficient to eliminate cancer cells, which is the reason why enhancing the function and quantity of CTLs may be of clinical benefit^14^.

Inhibitory receptors like CTLA-4, PD-1, TIM3 and LAG3, killer cell immunoglobulin-like receptor (KIR) and VISTA are co-expressed by T cells, secondary to chronic antigen stimulation^15^^-^^29^. This hyporesponsiveness makes cancer behave like a self-protein, inducing immune tolerance by transforming T-cell into an “exhaustion phenotype”^30^^-^^32^.

PD-1 receptor is expressed by activated T cells, NK cells, monocytes and B cells and binds PD-L1 (B7-H1 or CD274) and PD-L2 (B7-DC or CD273). PD-L1 is present in malignant cells as well as antigen presenter cells, myeloid cells, epithelial and lymphoid cells, and represents a constitutive or acquired mechanism of immune resistance^33^. PD-1/PD-L1 inhibit cytotoxic T lymphocyte proliferation, survival and effector activity, induce apoptosis of infiltrative T cells and increase the amount of regulatory T cells in the tumor microenvironment^34^.

## Clinical development of anti PD-1 and PD-L1 inhibitors in NSCLC

Currently, there are three PD-1/PD-L1 inhibitors available for lung cancer: nivolumab for squamous NSCLC (approved by FDA March 2015), pembrolizumab for the treatment of NSCLC and MPDL3280A from Roche (both granted breakthrough therapy designation) for NSCLC that has progressed on prior treatment (platinum based chemotherapy or targeted therapy for EGFR or ALK positive patients).

NSCLC was found to express PD-L1 in 27% to 57% of cases either in the cellular membrane or the cytoplasm^35^^,^^36^. PD-L1 and other molecules that act as immune checkpoints are up-regulated in response to substances secreted by lung tumor cells, such as the enzyme IDO which also down-regulates MHC-1^37^. Other immunosuppressive factors like IL-10 and TGF-β are secreted by tumor cells, editing the tumor microenvironment to attenuate immune response^38^. There are several clinical trials ongoing in lung cancer with different immune checkpoints blockers (**Tables 1****,****2**).

**Table 1:** Results of trials with PD-1 and PD-L1 inhibitors

Author	Indication	Compound	OS (m)	PFS (m)	ORR (%)	Phase
Lynch^39^ and Reck^40^	NSCLC and SCLC in 2^nd^ line	Ipilimumab	13	5	15	II
Zatloukal^41^	NSCLC in 2^nd^ line	Tremelimumab	−	−	5	II
Rizvi^42^ and Ramalingam^43^	Squamous cell in 2^nd^ line	Nivolumab	8.2	1.9	15	II
Rizvi^44^	NSCLC in 1^st^ line	Nivolumab	24	4	21	I
Brahmer^45^ and Gettinger^46^	NSCLC in 2^nd^ line	Nivolumab	8.2	−	18-25	I
Brahmer^47^	Squamous NSCLC in 2^nd^ line	Nivolumab	9.2 *vs*. 6	3.5 *vs*. 2.8	20 *vs*. 9	III
Paz-Ares^48^	Non squamous NSCLC in 2^nd^ line	Nivolumab	12.2 *vs*. 9.4	4.2 *vs*. 2.3	19 *vs*. 12	III
Antonia^49^	NSCLC in 1^st^ line	Nivolumab plus chemotherapy	16	−	33-47	I
Gettinger^50^ and Rizvi^51^	EGFR + (resistant to EGFR inhibitors)	Nivolumab plus erlotinib	OS 18 64%	29	15	I
Antonia^52^	NSCLC in 2^nd^ line	Nivolumab plus ipilimumab	OS 1-year 44%-65%	−	13-20	I
Antonia^53^	Recurrent SCLC after platinum	Nivolumab+/−ipilimumab	4.4 *vs*. 8.2	−	18 *vs*. 17	I
Garon^54^	NSCLC in 2^nd^ line NSCLC in 1^st^ line	Pembrolizumab	8	10; 27	21	I
Rizvi^55^	NSCLC PD-L1+ in 1^st^ line	Pembrolizumab	−	−	26	I
Ott^56^	SCLC PD-L1 + after failure of standard treatment	Pembrolizumab	−	Ongoing response	35	I
Patnaik^57^	NSCLC in 2^nd^ line	Pembrolizumab + ipilimumab	−	−	71	I
Brahmer^58^	NSCLC in 2^nd^ line	BMS-936559	−	−	10	I
Segal^59^	NSCLC in 2^nd^ line	MEDI4736	−	−	13	I
Khleif^60^	NSCLC in 2^nd^ line	MEDI4736			23	II
Soria^61^	NSCLC in 2^nd^ line	MPDL3280A	−	PFS 6-m 42%	23	I
Spira^62^	NSCLC in 2^nd^ and 3^rd^ line	MPDL3280A	11 *vs*. 9.5 m (ns); PD-L1 high (47 patients): NR *vs*. 11; PD-L1-: 9.7 *vs*. 9.7 m	−	15 *vs*. 15; high PD-L1: 38 *vs*. 13	II

**Table 2:** Current trials on anti PD-1 and anti PD-L1 inhibitors

Indication	Phase	Compound	Clinical trial No.
NSCLC, breast, pancreas	I	Nivolumab plus abraxane/CBDCA	02309177
Solid tumors	I	Nivolumab plus lirilumab-anti-KIR-	01714739
Solid tumors	I	Nivolumab plus BMS986016 –LAG3-	001968109
SCLC, breast	I	Nivolumab plus ipilimumab	01928394
NSCLC	I	Nivolumab plus ipilimumab or avastin or erlotinib or chemotherapy	01454102
NSCLC PD-L1+ 1^st^	III	Nivolumab	02041533
NSCLC 1^st^	III	Nivolumab *vs*. nivolumab plus ipilimumab *vs*. chemotherapy	02477826
SCLC 2^nd^	III	Nivolumab *vs*. chemotherapy	02481830
NSCLC in 1^st^ line	I	Pembrolizumab plus ipilimumab or chemotherapy or bevacizumab or erlotinib or gefitinib	02039674
PD-L1+ 2^nd^	I	Pembrolizumab	02007070
CNS 1^st^	II	Pembrolizumab	02085070
PD-L1+ 1^st^	III	Pembrolizumab	02142738
PD-L1+ 1^st^	III	Pembrolizumab	02220894
PD-L1+ 2^nd^	II and III	Pembrolizumab plus chemotherapy	01905657
EGFR+	I	Pembrolizumab plus afatinib	023646091
SCLC	II	Pembrolizumab	02359019
Solid tumors	I	MEDI0680 (AMP514)	02013804
Solid tumors	I	MEDI0680 (AMP514)	02118337
		MEDI4736	
NSCLC	I-II	MEDI4736 plus tremelimumab	02179671
Solid tumor	I-II	MEDI4736 plus tremelimumab	02000947
Solid tumor	I	MEDI4736	01693562
EGFR+ in 2^nd^ line	I	MEDI4736 plus gefitinib	02088112
EGFR+ in 2^nd^ line	I	MEDI4736 plus AZD9291	02143466
EGFR+ T790+ 2^nd^ line	III	MEDI4736 plus AZD9291 *vs*. AZD9291	02454933
NSCLC 1^st^ line	I-II	MEDI4736 plus sequential tremelimumab, gefitinib, AZD9291, selumetinib	02179671
Locally advanced or metastatic NSCLC	II	MEDI4736	01693562
Solid tumors	I-II	MEDI4736	01693562
Completely resected NSCLC	III	MEDI4736	02273375
NSCLC 3^th^	II	MEDI4736	02087423
NSCLC 1^st^ (EGFRwt, ALKwt)	III	MEDI4736 plus tremelimumab *vs*. MEDI4736 *vs*. chemotherapy	02455282
NSCLC 2^nd^	III	MEDI4736 (PD-L1+) *vs*. MEDI4736 plus tremelimumab (PD-L1-) *vs*. chemotherapy	02352948
Locally advanced or metastatic solid tumors: NSCLC, triple negative breast cancer, colorectal cancer	I	MPDL3280A (RG7446) plus Bevacizumab	01633970
NSCLC, melanoma, colorectal cancer	I	MPDL3280A (RG7446) plus cobimetinib	01988896
Locally advanced or metastatic PD-L1+ NSCLC 2^nd^ line	II	MPDL3280A (RG7446)	02031458
EGFR+ NSCLC	I	MPDL3280A (RG7446)T plus erlotinib	02013219
Non-squamous NSCLC in 2^nd^ line	III	MPDL3280A (RG7446)	02008227
Non-squamous NSCLC 1^st^	III	MPDL3280A *vs*. chemotherapy	02409355
Non-squamous 1^st^	III	MPDL3280A plus chemotherapy *vs*. chemotherapy	02367781 02409342
PD-L1+ NSCLC 1^st^ line	II	MPDL3280A (RG7446)	01846416
Solid tumors	I	MSB0010718C	01772004

### Anti PD-1 drugs

Nivolumab is a fully humanized anti-PD-1 monoclonal antibody from Bristol Meyer Squibb (BMS). A phase I trial in 2012 in previously treated NSCLC patients treated with intravenous nivolumab every 2 weeks for 12 cycles at different doses (0.1 to 10.0 mg per kilogram of body weight) demonstrated an overall response rate (ORR) of 18% by RECIST 1.1 criteria. These responses were seen in non-squamous as well as squamous NSCLC (ORR of 12% and 33%, respectively)^7^. Long term follow-up of the phase I trial with nivolumab in 129 previously treated NSCLC patients showed that in 50% of patients who responded, response was already evident by week 8 after starting treatment. In this trial, the ORR was 14% up to 25% and OS was 18% at 3 years. The median duration of response was 17 months. Pneumonitis was observed in 7% of patients, and grade 3-4 toxicities in 14%. The most common side effects were fatigue, diarrhea, and anorexia^63^.

The phase III CheckMate 017 trial reported an OS advantage for nivolumab in pretreated squamous NSCLC. It included 272 squamous NSCLC patients regardless of PD-L1 status. Patients were randomized to nivolumab (3 mg/kg i.v. in 60 min every 2 weeks) versus docetaxel. The OS endpoint was met early and the study was therefore stopped in January 2015. Median OS was 9.2 months in the nivolumab arm (95% CI: 7.3-13.3) and 6.2 months in the docetaxel arm (95% CI: 5.1-7.3). At 1-year, the OS was 42% for the nivolumab group versus 24% in the docetaxel group. The hazard ratio (HR) was 0.59 (95% CI: 0.44-0.79; *P*=0.00025). Nivolumab also improved median progression free survival (PFS) 3.5 *vs*. 2.8 months with docetaxel (HR =0.62; 95% CI: 0.47-0.81; *P*=0.0004) (**Table 1**)^47^.

CheckMate 063 assessed safety profile of nivolumab in squamous NSCLC. This was a phase II open label, multinational and multicenter single arm trial in 117 patients. Nivolumab was given to squamous NSCLC patients who had progressed to platinum based therapy and second line systemic chemotherapy. As with CheckMate 017, this trial included patients regardless of PD-L1 status. The most common adverse reactions were fatigue (50%), dyspnea (38%), musculoskeletal pain (36%), decreased appetite (35%), cough (32%), nausea (29%), and constipation (24%). There were two treatment-associated deaths caused by pneumonia and ischemic stroke that occurred in patients with multiple comorbidities and progressive disease. In 27% of patients, nivolumab was stopped due to severe adverse reactions. Seventeen out of 117 patients (ORR: 15%; 95% CI: 9-22) had partial responses with durability ranging from 1.9 to 11.5 months; 59% had responses of 6 months or longer^42^.

A phase III trial (CheckMate 057) randomized 582 patients with advanced non-squamous NSCLC after failing platinum doublet chemotherapy to nivolumab at 3 mg/kg i.v. every 2 weeks (*n*=292) or docetaxel (*n*=290). The study was stopped early after the primary endpoint of improved OS was reached. Median OS was 12.2 months with nivolumab *vs*. 9.4 months with docetaxel (HR =0.73; 95% CI: 0.59-0.89; *P*=0.00155), with a 1-year OS of 50.5% *vs*. 39.0% for docetaxel. ORR was 19% *vs*. 12% (*P*=0.0246). Fifty-two percent of the PD-1 inhibitor responses are still ongoing compared with 14% of the docetaxel responses^48^ (**Table 1**).

Results from a phase III trial CheckMate 026 comparing nivolumab versus chemotherapy in the first line setting for PD-L1 positive NSCLC patients are pending (NCT02041533).

There is also an ongoing phase I trial with multiple arms using combinations of nivolumab with chemotherapy, bevacizumab, ipilimumab or erlotinib (CheckMate 012, NCT01454102).

Now a phase III trial is planned to test the combination of nivolumab plus ipilimumab *vs*. chemotherapy in the first line setting (CheckMate 227, NCT02477826).

In small cell lung cancer (SCLC) a phase III trial will test nivolumab *vs*. topotecan in the second line setting (CheckMate 331, NCT02481830).

Preliminary results of platinum doublets combined with nivolumab have showed similar response rates when compared to chemotherapy alone in the first line setting (ORR: 33%-47%) and OS rate was 86% at 18 months in the nivolumab combined with carboplatin and taxol arm^49^.

Pembrolizumab (MK-3475) is an anti PD-1 monoclonal antibody from Merck Sharp & Dohme laboratory. Data from a phase Ib trial in previously treated patients showed an ORR of 21% by RECIST1.1 (26% in treatment naïve and 20% in previously treated patients). In treatment naïve patients, median PFS was 27 weeks, and median overall survival was not reached (OS at 6 months was 86%). In previously treated patients, median PFS was 10 weeks and OS 8.2 months. In the pooled population, median PFS was 13 weeks and 6-month PFS rate was 30%; median OS was 8.2 months with a 6-month OS rate of 64%. The drug was well tolerated with grade 2-4 adverse events in 10% of cases, most commonly pneumonitis^54^^,^^64^. In view of these results, pembrolizumab (formerly known as lambrolizumab) was designated as breakthrough therapy for lung cancer treatment by the FDA.

Currently pembrolizumab is being compared with docetaxel in a phase II/III trial in advanced PD-L1 positive NSCLC (NCT01905657). Pembrolizumab is administered every 3 weeks at two different doses (2 mg/kg i.v. every 3 weeks and 10 mg/kg i.v. every 3 weeks) *vs*. docetaxel (75 mg/m i.v. every 3 weeks).

A phase I trial in PD-L1 positive NSCLC with pembrolizumab (NCT02039674) is currently ongoing, as is a phase I/II trial with ipilimumab or chemotherapy in combination with pembrolizumab (NCT02039674) and a phase II trial of adjuvant pembrolizumab after chemo-radiotherapy for stage III NSCLC patients (NCT02343952) (**Table 2**).

In addition to the above mentioned trials, two phase III trials of pembrolizumab at a fixed dose of 200 mg i.v. every 3 weeks for up to 35 treatments is also being compared in the first line setting *vs*. platinum based chemotherapy in PD-L1 positive and PD-L1 strong positive NSCLC patients (Keynote 042, NCT02220894 and Keynote 024, NCT02142738) (**Table 2**).

Data in SCLC patients were presented in May 2015 as preliminary results of an ongoing multi-cohort, phase Ib study of pembrolizumab in patients with PD-L1+ advanced solid tumors (Keynote 028). The SCLC cohort had an ORR of 35% with durable responses^56^ (**Table 1**).

An ongoing phase II trial is testing pembrolizumab in patients with extensive stage small cell lung cancer after completion of combination chemotherapy (NCT02359019) (**Table 2**).

### Anti PD-L1 drugs

BMS-936559 is a fully humanized IgG4 monoclonal antibody against PD-L1. This anti PD-L1 was used in 207 patients with advanced stage solid tumors, 75 of whom had NSCLC. The results of the study showed an ORR close to 10% and stable disease for up to 6 months in 18% patients^58^. However, further development of this drug has been halted by Brystol Meyer Squibb (**Table 1**).

MEDI4736 (Astra Zeneca) is a monoclonal antibody designed with a mutated FC domain in order to prevent antibody-dependent cell mediated cytotoxicity (ADCC). Preliminary results of a phase I trial in patients with different solid tumor types including NSCLC reported clinical benefit and durable disease control with no dose limiting toxicities or grade 3-4 toxicities^60^. Objective response was seen in 23% of patients with pretreated NSCLC (12 out of 53 evaluable patients) in the phase II trial^60^. Results reported at the 2014 ASCO annual meeting showed the drug was well tolerated at all tested doses. Doses were escalated from 0.1 to 10 mg/kg every 2 weeks, with extension to 15 mg/kg every 3 weeks^65^. Pneumonitis, hyperglycemia and colitis were not reported.

Preliminary results from an ongoing study with 346 patients with solid tumors, of whom 143 had NSCLC, used MEDI4736 at dosages of 10 mg/kg every 2 weeks for 1 year. Only 6% of patients had grade 3-4 drug related serious adverse events. The median treatment duration was 8 weeks, and activity was seen as early as 6 weeks. After finishing active therapy, ORR in NSCLC was 13%^59^.

Adjuvant MEDI4736 after chemo-radiotherapy for unresectable stage III NSCLC is currently being tested in a phase III trial (10 mg/kg i.v. every 2 weeks) (NCT02125461). Safety of doses of 0.1-10 mg/kg every 2 weeks or 15 mg/kg every 3 weeks is also been evaluated in a phase I trial in advanced solid tumors including NSCLC (NCT01693562). The anti CTLA-4 IgG2 monoclonal antibody tremelimumab is also being tested in combination with MEDI4736 in a phase Ib trial in NSCLC. (NCT02000947), and a phase III trial in the first line setting will compare the combination with tremelimumab *vs*. MEDI4736 as single agent *vs*. chemotherapy in advanced NSCLC according to PD-L1 status (**Table 2**).

For EGFR positive patients with T790 mutation, a phase III trial is comparing AZD9291 as single agent *vs*. combination with MEDI4736 (NCT02143466). Other ongoing phase I trials are combining small molecules with MEDI4736 (NCT02179671, NCT02143466). There is currently a phase II trial recruiting PD-L1 positive patients who have received at least two prior systemic treatment regimens including one platinum-based chemotherapy regimen to receive MEDI4736 (NCT02087423) (**Table 2**).

Atezolizumab MPDL3280A from Hoffman la Roche is also an anti-PD-L1 monoclonal antibody, IgG type with Fc domain engineered modification to avoid ADCC. Preliminary phase I results in previously treated advanced NSCLC patients reported a response rate of 23% in squamous and non-squamous NSCLC, 24-week PFS was 48% and median time to response was 12 weeks^61^^,^^66^. Results from the phase II POPLAR study presented at the 2015 ASCO annual meeting showed that atezolizumab improved median OS compared with docetaxel in previously treated patients with PD-L1 strong positive NSCLC. For the 47 patients with high PD-L1 expression from 277 included in the study, median OS was not reached in those treated with atezolizumab *vs*. 11 months in patients treated with docetaxel (HR =0.46; 95% CI: 0.19-1.09). In high expression PD-L1 patients, ORR was 38% with atezolizumab *vs.* 13% with chemotherapy^62^ (**Table 1**).

The FIR trial is completed and results are pending. This is a phase II study using this drug in the first setting of PD-L1 positive NSCLC patients (NCT01846416). Other ongoing trials are a phase I study assessing the combination of erlotinib and MPDL3280A in EGFR mutated adenocarcinoma (NCT02013219), a phase III trial where MPDL3280A is being tested at a dose of 1,200 mg i.v. every 3 weeks *vs*. docetaxel at dose of 75 mg/m^2^ i.v. every 3 weeks after chemotherapy failure (NCT02008227), a phase III trial in the first line setting in non-squamous NSCLC (NCT02409355) and three phase III trials testing the combination of MPDL3280A in the first line setting of non-squamous NSCLC (NCT022367781, NCT02409342, NCT02367794).

### Anti CTLA-4 antibodies

Ipilimumab (anti CTL-4 antibody) is a fully humanized Ig G1 monoclonal antibody approved for metastatic melanoma. It was tested in a phase II trial in 334 lung cancer patients (204 NSCLC and 104 SCLC patients) in the first line setting. The trial featured two ipilimumab arms in combination with chemotherapy (paclitaxel plus carboplatin), one arm in a phased schedule and the other one in concomitant schedule *vs*. a third arm of chemotherapy alone. Median PFS was longer with the phased combination (5.1 *vs*. 42 months; HR =0.69; 95% CI: 0.48-1.00; *P*=0.02). Subgroup analysis showed higher activity in squamous cell lung cancer. Toxicity was moderate with grade 3-4 toxic effects in 15% of patients^39^. A phase III trial with ipilimumab in combination with chemotherapy in a phased schedule in squamous NSCLC was completed and results are pending (NCT01285609). Ipilimumab is also being tested in SCLC (NCT01331525, NCT01450761, and NCT02046733) and in combination with ALK and EGFR inhibitors (NCT01998126).

Tremelimumab is a monoclonal antibody similar to ipilimumab and is developed simultaneously. Although tremelimumab is similar to ipilimumab, the pivotal trail in advanced melanoma was negative, and this was the reason why clinical development of the drug was stopped during years. Tremelimumab was also tested in NSCLC in a phase II study including pre-treated patients. Patients were randomized to tremelimumab or best supportive care after four cycles of a platinum combination. The response rate was poor, just 5%, and there were no differences in PFS^41^.

This drug is now being tested in combination with the target drug gefitinib (NCT02040064), with the anti PD-L1 MEDI4736 (NCT02000947, NCT02179671) and with the OX-40 agonist MEDI6469 (NCT02205333) (**Table 2**).

## PD-L1 immunohistochemical expression and response to therapy

Immunohistochemistry (IHC) has been used to measure PD-L1 expression in cancer cells, as well as in tumor infiltrating lymphocytes^67^. Interpretation of outcomes is complicated due to the range of techniques and because there are different antibodies used by every pharmaceutical company. Also, timing of the biopsy is a variable that can affect results since expression of the PD-L1 changes during tumor evolution^68^^,^^69^. The cut-off at which PD-L1 positivity is considered positive, is an important factor for the interpretation of results. For example, 1% of cut-off has been used in studies with pembrolizumab and depending on this percentage, negativity, light positivity or intense positivity has been defined. Taking into account these different cut-off values, there is a reported 30% of intense positivity of PD-L1 for NSCLC. In the reported studies with nivolumab, a 5% of membrane staining of tumor cells was considered as positive. About 33%-48% of tumor samples were PD-L1 positive in the nivolumab studies. In the studies of MPDL3280A, PD-L1 positivity criteria included 5% of IHC staining on tumor infiltrating lymphocytes and tumor cells. According to these criteria, 25% of NSCLC samples were positive for PD-L1 expression^66^.

Further studies are required to demonstrate if PD-L1 expression by IHC correlates with a higher response rate when tumor cells are positive for staining. Median response rate of 38% in PD-L1 positive patients (ranging from 23% to 83%) *vs*. 7% (ranging from 0%-15%) in PD-L1 negative patients have been seen^54^^,^^59^^,^^60^^,^^67^^,^^68^^,^^70^^-^^72^.

A pembrolizumab trial, Keynote 001, has shown improved ORR in patients with positivity for PD-L1 expression. A total of 495 patients were assigned to receive pembrolizumab (at a dose of either 2 or 10 mg per kilogram of body weight every 3 weeks or 10 mg per kilogram every 2 weeks) to either a training group (182 patients) or a validation group (313 patients). Results of PD-L1 expression were reported as the percentage of neoplastic cells with PD-L1 membrane staining; objective responses among all patients was 19.4%, and the median duration of response was 12.5 months. The median PFS was 3.7 months, and median OS was 12.0 months. Cut-off for the training group was PD-L1 expression in at least 50% of tumor cells. A response rate of 45.2% was seen among patients with a proportion score of at least 50% in the validation group, and, among patients with a proportion score of at least 50%; median PFS was 6.3 months while median OS was not reached^68^^,^^72^. Other studies as the study from Garon *et al*.^72^ reported higher response rate and longer survival for PD-L1 positive cases.

In other types of tumors like melanoma, the predictive value of PD-L1 can be different, as the response rate in PD-L1 negative cases is higher than in PD-L1 negative lung cancer cases^73^. In melanoma, PD-L1 expression, as well as the presence of infiltrating CD8 lymphocytes, has been described as a better predictive factor for response to anti PD-1 drugs than considering only the PD-L1 expression^74^.

## Synergistic combinations and innovative approaches

Synergy can be achieved by targeting different immune checkpoints with combinations, as combining antibodies anti checkpoints with target drugs, antiangiogenic drugs or chemotherapy.

After promising activity in melanoma was reported, trials in lung cancer demonstrated that the combination of a PD-1 inhibitor and anti CTLA-4 antibody was active, with an ORR of 13%-20%^52^. Several ongoing trials are testing different combinations in lung cancer, including several phase III trials (**Table 2**).

In a phase I/II clinical trial (CheckMate 032), nivolumab was studied with or without ipilimumab for treatment of recurrent SCLC. This open-label study randomized patients to nivolumab 3 mg/kg i.v. every 2 weeks or nivolumab plus ipilimumab (1+1, 1+3 or 3+1 mg/kg) i.v. every 3 weeks for four cycles, followed by nivolumab 3 mg/kg every 2 weeks. ORR was 18% with nivolumab and 17% with nivolumab/ipilimumab. Median OS was 4.4 months with nivolumab monotherapy and 8.2 months with combination therapy^53^ (**Table 1**).

Interim results from a phase I study evaluating pembrolizumab plus ipilimumab in patients with recurrent NSCLC reported an ORR of 71%, showing a decrease in target lesion burden^57^ (**Table 1**).

Another possible combination is MAPK pathway inhibitors. In the past two decades, targeted therapies in NSCLC, mainly EGFR inhibitors such as erlotinib, gefitinib, afatinib and ALK and ROS-1 inhibitors like crizotinib, have developed rapidly and have shown high response rates^75^. The possible combinations with immunotherapy could presumably allow long lasting effects by means of enhanced tumor antigen presentation by dendritic cells after apoptosis, necrosis and immune checkpoint blockade, allowing infiltrating cytotoxic T cells to attack tumor cells^76^.

Akbay *et al*.^77^ showed that in murine lung tumor cells there is upregulation of tumor PD-L1 via mutant EGFR signaling, and that in preclinical models there was a survival benefit when therapeutic blockade of the PD-L1 pathway was attempted.

A study analyzing biopsies from 123 NSCLC patients reported 56 EGFR mutant and 29 KRAS mutant tumors, with a significant correlation of EGFR mutation with PD-L1 expression, and KRAS mutation with PD-1 expression. Moreover, EGFR positive patients treated with EGFR inhibitors had better survival when they had PD-L1 molecule expression. In a phase I trial, erlotinib plus nivolumab showed a median PFS of 29 months, an OS rate of 64% at 18 months and ORR of 15%^78^.

Trials testing the combination of erlotinib, gefitinib, afatinib or novel anti EGFR drugs plus ipilimumab, nivolumab, MPDL3280A and tremelimumab are currently ongoing. Also a phase I trial is ongoing testing the combination for ALK positive of ceritinib and nivolumab tumors (NCT02393625) (**Table 2**).

Several trials are currently assessing different aspects of the combination of antiangiogenic and immunotherapy, including a phase I trial evaluating the safety and tolerability of nivolumab as maintenance therapy in combination with bevacizumab in NSCLC (NCT01454102) (**Table 2**).

With regard to combinations with chemotherapy, ipilimumab was studied in a phase III trial with dacarbazine for advanced melanoma. OS was significantly longer in the arm receiving ipilimumab plus dacarbazine than in the group receiving dacarbazine plus placebo (11.2 *vs*. 9.1 months; HR for death =0.72; *P*<0.001)^79^. Ipilimumab is currently being studied in combination with lenalidomide for advanced or metastatic cancers with no available standard therapy (NCT01750983). As we have previously mentioned, ipilimumab was studied in a phase II trial in chemotherapy naïve patients with NSCLC. Patients were randomly assigned to receive paclitaxel and carboplatin with either placebo or concurrent ipilimumab or phased ipilimumab. The study met its primary end point of improvement in terms of immune-related progression-free survival (irPFS) for phased ipilimumab *vs*. the control (HR =0.72; *P*=0.05), but not for the concurrent arm (HR =0.81; *P*=0.13)^39^. Nivolumab has also been studied in a phase I trial in combination with carboplatin and nab-paclitaxel in stage IIIB and IV NSCLC (NCT02309177). There is also a phase I multi-arm study of nivolumab in combination with gemcitabine/cisplatin, pemetrexed/cisplatin, carboplatin/paclitaxel, bevacizumab maintenance, erlotinib, ipilimumab or as monotherapy in subjects with stage IIIB/IV NSCLC (NCT02309177) (**Table 2**).

Radiation therapy has been described to induce tumor regression in non-irradiated sites, and this rare phenomenon is called the “abscopal effect”. There was a case of a melanoma patient treated with ipilimumab and radiotherapy; the patient had a systemic response to localized radiotherapy after progressing on ipilimumab. Non-irradiated areas also regressed and disease remained stable and minimal months later^80^. Radiotherapy has been shown to enhance antigen presentation by myeloid cells within the tumor microenvironment, therefore increasing T-cell killing of the malignant cells, a mechanism which is thought to mediate the abscopal effect. Combinations of radiotherapy with immune checkpoint inhibitors are currently being studied. For example, there is an ongoing phase II trial of ipilimumab with stereotactic body irradiation in advanced lung cancer (NCT02239900), pembrolizumab with hypofractionated stereotactic radiation therapy (NCT02444741), and pembrolizumab with concurrent chemo-radiation for SCLC (NCT02402920).

## Toxicity

When combining ipilimumab with PD-1 inhibitors like nivolumab in melanoma, drug-related adverse events of grade 3 or 4 were reported in 53% of patients compared with 18% of patients who received ipilimumab monotherapy^72^^,^^81^^,^^82^. Grade 3 or 4 adverse events, regardless of attribution, were observed in 72% of patients, and grade 3 or 4 treatment-related events were noted in 53%, with the most common events being elevated levels of lipase (in 13% of patients), aspartate aminotransferase (in 13%), and alanine aminotransferase (in 11%). A total of 6 in 28 patients (21%) had grade 3 or 4 treatment-related events that were dose-limiting.

Even though ipilimumab is generally well tolerated, severe immune mediated side effects have been observed including enterocolitis, hepatitis, dermatitis, neuropathies, and endocrinopathies. These reactions can manifest during treatment or even months after discontinuation of ipilimumab. In the phase III study of ipilimumab, enterocolitis was the most common severe toxicity, seen in 34 of 511 patients. One percent of patients had bowel perforation and 0.8% died^83^^,^^84^. Treatment of severe reactions mainly consists of discontinuation of ipilimumab and initiation of systemic corticosteroids at a dosage of 1 to 2 mg/kg per day of prednisone or equivalent with taper over a period of 1 month once the toxicities have considerably improved^85^.

Nivolumab is a better tolerated drug. The most common adverse reactions (≥20%) reported in a phase III clinical trial were fatigue (50%), dyspnea (38%), musculoskeletal pain (36%), decreased appetite (35%), cough (32%), nausea (29%), and constipation (24%). Only 6% of grade 3 or 4 toxicities are seen. Skin toxicity, including rash, pruritus, and vitiligo are the most commonly seen reactions (31% of the cases). Diarrhea was seen in 11% of cases and pneumonitis in 3%. Transaminitis was seen in 11% and thyroid abnormalities in 3%. Treatment of severe reactions consists of withdrawal of the drug and, if required, prednisone 1 to 2 mg/kg daily should be given until the patient is back at baseline and then tapered over a month^86^.

With pembrolizumab, 13% of patients experienced grade 3 or 4 drug-related adverse events. Common low grade reactions were fatigue, asthenia, fever, chills, myalgias and headaches. Pneumonitis was reported in 4% of cases and grade 3 transaminitis. Toxicities improved after pembrolizumab discontinuation and corticosteroid therapy. Only one case of grade 3 diarrhea was reported, which improved without steroids, and 8% of hypothyroidism which improved with thyroid hormone replacement^72^.

## Other immunomodulatory and agonistic molecules

Other immune checkpoint antibodies are currently been studied as single agents, and more interestingly, in combination with other checkpoint inhibitors. The most relevant immune checkpoints currently under clinical investigation are lymphocyte activation gene -3 (LAG-3), KIR, OX 40, GITR, and 4-1BB.

As for LAG-3, preclinical studies have shown co-expression of LAG-3 and PD-1 on tumor infiltrating cells. Combinations of anti-LAG-3 antibodies with anti PD-1 antibodies showed decreased tumor growth and enhanced antitumor immunity, and also maintained immune homeostasis with decreased autoimmune responses^87^. There is currently an ongoing phase I trial testing anti-LAG-3 in advanced NSCLC (NCT01968109).

KIR is a molecule in the NK cell that binds HLA molecules to the surface of tumoral cells, inhibiting NK lymphocyte cytotoxic activity against malignant cells. If KIR is blocked, then NK cells are unblinded and can recognize and attack tumoral cells^88^. Lirilumab is an anti-KIR antibody currently in phase I trials in NSCLC (NCT01750580).

GITR affects regulatory T cells (Tregs) and also acts as a costimulatory receptor expressed after T cell activation that enhances T cell function and survival. Treatment with GITR agonistic antibody destabilizes intra-tumor Tregs allowing for more efficient cytolysis by CD8^+^ T cells^89^. A phase I trial with anti-GITR antibody TRX-518 is ongoing in advanced solid tumors (NCT01239134).

4-1BB, also known as CD137 receptor, a member of the TNF family of receptors, is an immunomodulatory molecule expressed in immune cells. Urelumab (BMS 663513) is a CD137 agonist that has been tested in a phase I/II study with promising activity, although marked hepatic toxicities have been reported. A study of urelumab in combination with chemo-radiotherapy for NSCLC has been terminated (NCT00461110) and the combination with nivolumab is being tested in solid tumors and B cell non-Hodgkin lymphoma (NCT02253992)^90^. There is an ongoing trial with 4-1BB agonist PF-05082566 plus PD-1 inhibitor MK-3475 in patients with solid tumors (NCT02179918)^90^.

OX40 promotes T cell survival and expansion. Preclinical studies showed that OX40 agonists increase anti-tumor immunity and improve disease free survival. Patients treated with one course of the antiOX40 mAb showed regression of at least one metastatic lesion in 12/30 patients and an acceptable toxicity profile^91^.

## Cancer vaccines

Two types of vaccines, antigen specific and cell-based, are currently being studied in lung cancer.

### Antigen specific vaccines

Several vaccines target tumor specific antigens, as those against melanoma-associated antigen-A3 (MAGE-3), MUC-1, EGFR, human telomerase reverse transcriptase (hTERT) or NY-ESO-1.

MAGE-A3: 35%-42% of NSCLCs have expression of MAGE-A3, which is presented to T cells. A phase II trial showed a correlation between the expression of a gene signature and immune related transcripts associated with better outcome when a MAGE A3 vaccine was used in NSCLC^92^.

L-BLP25 (stimuvax) targets the peptide MUC1 which is overexpressed in lung cancer and associated with poor prognosis^93^. Early trials with L-BLP25 were promising but the phase III trial did not meet its primary endpoint of OS^94^. Two studies are currently ongoing to evaluate L-BLP25, one in combination with bevacizumab for inoperable patients with stage III-IV NSCLC following chemo/radiotherapy (NCT00828009), and another in Asian patients is pending results (NCT01015443).

rEGF: 40%-80% of NSCLCs express EGFR. Therefore a vaccine composed of humanized recombinant EGF is being studied. Two trials were started but terminated to initiate a new phase III with a biomarker to enrich the population. Median OS was 11.7 months for patients with anti-EGF antibody response *vs*. 3.6 months for non-responders^95^. A phase III trial is now planned (NCT01444118).

Anti-telomerase-based vaccine GV1001 showed clinical benefit in a phase I and II trial, with PFS improvement in inoperable NSCLC after chemoradiation (19 months in responders *vs*. 3.5 months in non-responders, *P*=0.001). Responders were those who developed GV1001-specific T cell memory responses and had IFN-γ (high)/IL-10(low)/IL-4(low) cytokine profiles^96^.

An NY-ESO-1 vaccine achieved antibody responses in 9 of 10 patients. Of these 10, 2 patients with lung cancer and 1 with esophageal cancer showed stable disease^97^. A clinical trial in cancer patients with tumors expressing NY-ESO-1 tested the vaccine with or without sirolimus (NCT01522820).

### Cell based vaccines

Belagenpumatucel-L (Lucanix) is composed of five transforming growth factor B2 (TGF-B2) antisense gene-modified allogenic NSCLC cell lines. In a phase III clinical trial for those patients that were included after completing 12 weeks of chemotherapy, survival analysis showed improvement in overall survival (20.7 *vs*. 13.4 months, *P*=0.8) and for those patients treated with radiotherapy, median survival was 40 *vs*. 10 months (*P*=0.036)^98^.

Tergenpumatucel-L consists of genetically modified allogeneic NSCLC tumor cells lines with the alpha-(1,3)-galactosyltransferase (αGal) moiety on the cell surfaces which generates an innate immune reaction, killing the foreign NSCLC tumor cells. In a phase II clinical trial, 28 patients with metastatic NSCLC or recurrent disease received the vaccine with a median OS of 11.3 months and a long stabilization in 8 of 28 patients with one patient alive after 50 months of follow-up. Patients who received salvage chemotherapy after progressing on tergenpumatucel-L had a better OR to subsequent chemotherapy treatments than patients who had not received prior tergenpumatucel-L^99^.

Peptide vaccines have been also tested in solid tumors, such as the peptide vaccine against the indoleamine 2,3-dioxygenase (IDO) enzyme. In a phase I trial in 15 advanced NSCLC, HLA-A2 positive patients, one patient developed a partial response after 1 year of treatment with the vaccine, and long-lasting stable disease was demonstrated in further six patients. Median OS was 25.9 months. Furthermore, expression of IDO was detected in nine of ten tumor biopsies by IHC. In long-term analyses of two clinically responding patients, the ratio of kynurenine/tryptophan in serum (Kyn/Trp) remained stable^100^.

## Conclusion

New strategies to treat lung cancer, inducing durable responses to therapy, are currently being developed. The incorporation of immunotherapy to the arsenal of drugs for treatment of lung cancer is a promising approach to reach these goals, with low rates of side effects.

Personalized medicine currently offers the best profile in terms of side effects and efficacy in different cancers, and combination with immune checkpoint blockers could enhance its activity.

In view of promising results reported, now the challenge is finding those biomarkers that can help us select the best treatment approach for every patient.
